# Body Mass Index, Interleukin-6 Signaling and Multiple Sclerosis: A Mendelian Randomization Study

**DOI:** 10.3389/fimmu.2022.834644

**Published:** 2022-03-21

**Authors:** Marijne Vandebergh, Sara Becelaere, CHARGE Inflammation Working Group, Bénédicte Dubois, An Goris

**Affiliations:** ^1^ Laboratory for Neuroimmunology, Department of Neurosciences, Leuven Brain Institute, KU Leuven, Leuven, Belgium; ^2^ Laboratory for Human Evolutionary Genetics, Department of Human Genetics, KU Leuven, Leuven, Belgium; ^3^ Department of Neurology, University Hospitals Leuven, Leuven, Belgium

**Keywords:** Mendelian randomization, multiple sclerosis, obesity, interleukin-6, c-reactive protein, genetic epidemiology, susceptibility

## Abstract

**Objectives:**

We explored whether genetically predicted increased body mass index (BMI) modulates multiple sclerosis (MS) risk through interleukin-6 (IL-6) signaling.

**Methods:**

We performed a two-sample Mendelian randomization (MR) study using multiple genome-wide association studies (GWAS) datasets for BMI, IL-6 signaling, IL-6 levels and c-reactive protein (CRP) levels as exposures and estimated their effects on risk of MS from GWAS data from the International Multiple Sclerosis Genetics Consortium (IMSGC) in 14,802 MS cases and 26,703 controls.

**Results:**

In univariable MR analyses, genetically predicted increased BMI and IL-6 signaling were associated with higher risk of MS (BMI: odds ratio (OR) = 1.30, 95% confidence interval (CI) = 1.15-1.47, *p* = 3.76 × 10^-5^; IL-6 signaling: OR = 1.51, 95% CI = 1.11-2.04, *p* = 0.01). Furthermore, higher BMI was associated with increased IL-6 signaling (β = 0.37, 95% CI = 0.32,0.41, *p* = 1.58 × 10^-65^). In multivariable MR analyses, the effect of IL-6 signaling on MS risk remained after adjusting for BMI (OR = 1.36, 95% CI = 1.11-1.68, *p* = 0.003) and higher BMI remained associated with an increased risk for MS after adjustment for IL-6 signaling (OR = 1.16, 95% CI =1.00-1.34, *p* = 0.046). The proportion of the effect of BMI on MS mediated by IL-6 signaling corresponded to 43% (95% CI = 25%-54%). In contrast to IL-6 signaling, there was little evidence for an effect of serum IL-6 levels or CRP levels on risk of MS.

**Conclusion:**

In this study, we identified IL-6 signaling as a major mediator of the association between BMI and risk of MS. Further explorations of pathways underlying the association between BMI and MS are required and will, together with our findings, improve the understanding of MS biology and potentially lead to improved opportunities for targeted prevention strategies.

## Introduction

Multiple sclerosis (MS) is a complex autoimmune disease of the central nervous system (CNS), with both genetic variants and lifestyle/environmental factors involved in disease susceptibility (1). As modification of environmental and lifestyle factors offers potential for disease prevention, it is important to pinpoint causal links between these factors and MS.

Mendelian randomization (MR) analyses, in which genetic variants are used as a proxy for environmental/lifestyle exposures, help overcome limitations of observational studies, i.e., reverse causation, recall bias and residual confounding. Hence, they are an elegant tool to strengthen causal inference. MR analyses have consistently shown a causal relation between lower 25-hydroxyvitamin D (25OHD) levels, higher body mass index (BMI) and an increased risk for MS (2–12), in line with observational studies (13–18). Previously, it has been demonstrated that 25OHD and BMI act largely independently on risk of MS, with approximately 5% of the effect of BMI on MS mediated by 25OHD (11). In addition, leptin and adiponectin, proposed mediators of the BMI-MS association, did not show an effect on the risk of MS (11). Therefore, the majority of the effect of obesity remains unexplained.

Observational studies have shown that serum concentrations of interleukin-6 (IL-6), an adipose-associated cytokine, are influenced by BMI (19). As patients with obesity suffer from a chronic low-grade pro-inflammatory state (20) in which plasma levels of pro-inflammatory cytokines such as IL-6 are elevated (21), we explored whether IL-6 signaling mediates the association between BMI and risk of MS.

For this purpose, through two-sample MR we first investigated the contribution of BMI to IL-6 signaling and of IL-6 signaling to risk of MS. In an MR mediation analysis, we determined to which extent IL-6 signaling mediates the association between BMI and risk of MS.

## Materials and Methods

### Data Sources

Data sources for adult body mass index (BMI), interleukin-6 (IL-6) signaling, serum IL-6 levels, c-reactive protein (CRP) levels and multiple sclerosis (MS) risk are summarized in **Supplementary Table 1**.

Genome-wide significant (p < 5 × 10^-8^) genetic variants for BMI were extracted from, to date, the largest meta-analysis genome-wide association study (GWAS) for BMI from the Genetic Investigation of Anthropometric Traits (GIANT) consortium, in n = 681,275 individuals of European ancestry (22). We selected 656 primary associations, as described in a previous study from our group (10) (**Supplementary Table 2**). In secondary analyses, we selected 77 BMI SNPs identified in individuals of European ancestry not including the UK Biobank cohort (23) (**Supplementary Table 3**).

Genetic variants for IL-6 signaling were selected as uncorrelated (*r^2^* < 0.1) SNPs within 300 kb of the IL-6 receptor gene (*IL-6R*, GRCh37/hg19 coordinates: chr1:154077669-154741926) that are associated with CRP in the UK Biobank (24) (GWAS round 2, n = 343,524 individuals) at genome-wide significance (**Supplementary Table 4**).

Additionally, uncorrelated (*r^2^* < 0.1) genetic variants genome-wide significantly associated with CRP within 300 kb of *IL-6R* in the Cohorts for Heart and Aging Research in Genomic Epidemiology (CHARGE) Inflammation Working Group GWAS of 204,402 individuals of European ancestry were selected (25), as described in Georgakis et al. (26) (**Supplementary Table 5**).

CRP is a relevant downstream biomarker for IL-6 signaling. It has been shown previously that genetic variants reflecting higher levels of CRP and thus increased IL-6 signaling correlate with lower soluble IL-6 receptor (IL-6R) levels and lower circulating IL-6 levels (26, 27).

SNPs reaching genome-wide significance for circulating serum IL-6 levels in the combined GWAS meta-analysis from the CHARGE Inflammation Working Group of 67,428 individuals of European ancestry were selected (28) (**Supplementary Table 6**).

Finally, SNPs associated with CRP levels at genome-wide significance throughout the genome and clumped at *r^2^* < 0.1 were selected from the CHARGE Inflammation Working Group of 67,428 individuals of European ancestry (25) for trans-MR analysis (**Supplementary Table 7**). From these SNPs, SNPs were selected within 300kb of the *CRP* gene (chr1: 159,382,079-159,984,379) for cis-MR (**Supplementary Table 8**).

Genetic estimates for MS susceptibility were derived from the discovery cohorts of the latest IMSGC meta-analysis, including up to 41,505 participants (14,802 MS and 26,703 controls) (29). We selected the 138 primary, independent non-MHC SNPs for bi-directional MR (**Supplementary Table 9**).

### Selection of Instrumental Variables

Clumping and data harmonization were implemented in R v3.6.1 using the TwoSampleMR package (RRID: SCR_019010, v0.5.6) (30). SNPs were excluded from analyses if their measured linkage disequilibrium (LD) is *r^2^* > 0.05 in the European samples of 1000 Genomes. Furthermore, palindromic SNPs were replaced by non-palindromic proxy SNPs in high LD (*r^2^* ≥ 0.9) if the forward strand alleles could not be inferred based on allele frequencies. For exposure-associated variants not directly ascertained in the outcome datasets, we looked for proxy SNPs in high linkage disequilibrium (*r^2^* ≥ 0.9) using the LDlinkR package v1.1.2 in R v4.0.2.

An overview of the instrumental variables included in each MR analysis is provided in **Supplementary Tables 10–29**.

### Statistical Analyses

Univariable MR analyses were implemented in R v3.6.1 using the TwoSampleMR package (RRID: SCR_019010, v0.5.6) (30). When both marginal and conditional effect estimates were reported in the original studies, the marginal effect estimates were used in two-sample MR analyses.

As primary analysis, the multiplicative random-effects inverse-variance weighted (IVW) analysis was used to estimate the effects of the instrumental variables on outcomes (30, 31). The Cochran Q test and *I^2^* statistic (32) were calculated to measure the degree of heterogeneity across the individual effect estimates derived from each genetic variant (33).

Additional sensitivity tests were performed, including MR Egger (34), weighted median regression (35) and simple and weighted mode-based estimator (36). Horizontal pleiotropy was evaluated based on the intercept obtained from the MR Egger analysis being significantly different from 0 (34, 37) and by visual inspection of the funnel plot, where asymmetry is indicative of horizontal pleiotropy (30). Furthermore, we used the MR Pleiotropy RESidual Sum and Outlier (MR-PRESSO) method for detection of horizontal pleiotropy (MR-PRESSO global test) and correction of horizontal pleiotropy, if detected, *via* outlier removal (MR-PRESSO outlier test) (38). In MR-PRESSO analyses with BMI as exposure, we increased the number of simulations to compute the null distribution (NbDistribution) to 10,000 (default = 1,000). In addition, sensitivity tests were applied in which we excluded variants within the major histocompatibility complex (MHC) region, if present, as it is strongly associated with MS risk and susceptible to bias from pleiotropy due to its complex LD patterns.

We considered as MR results indicative of causal effects those that were concordant in direction across multiple MR approaches and with *p* value < 0.05 in IVW MR. The MR sensitivity analyses were only performed to explore the robustness of the main IVW analysis to potential pleiotropy and as such no statistic sensitivity threshold was applied for these.

Multivariable MR (MVMR) analyses were implemented in R v3.6.1 using the TwoSampleMR package (RRID: SCR_019010, v0.5.6) (30) for the inverse-variance weighted method. Additional sensitivity analyses robust to pleiotropy, i.e. MVMR-Egger, MVMR-Robust, MVMR-Median and MVMR-Lasso, and robust to weak instruments, i.e. MVMR-Q(het) were implemented from the MendelianRandomization R package (39), the MVMR R package (40) and from the R code described in Grant et al. (41).

**Figure 1:**
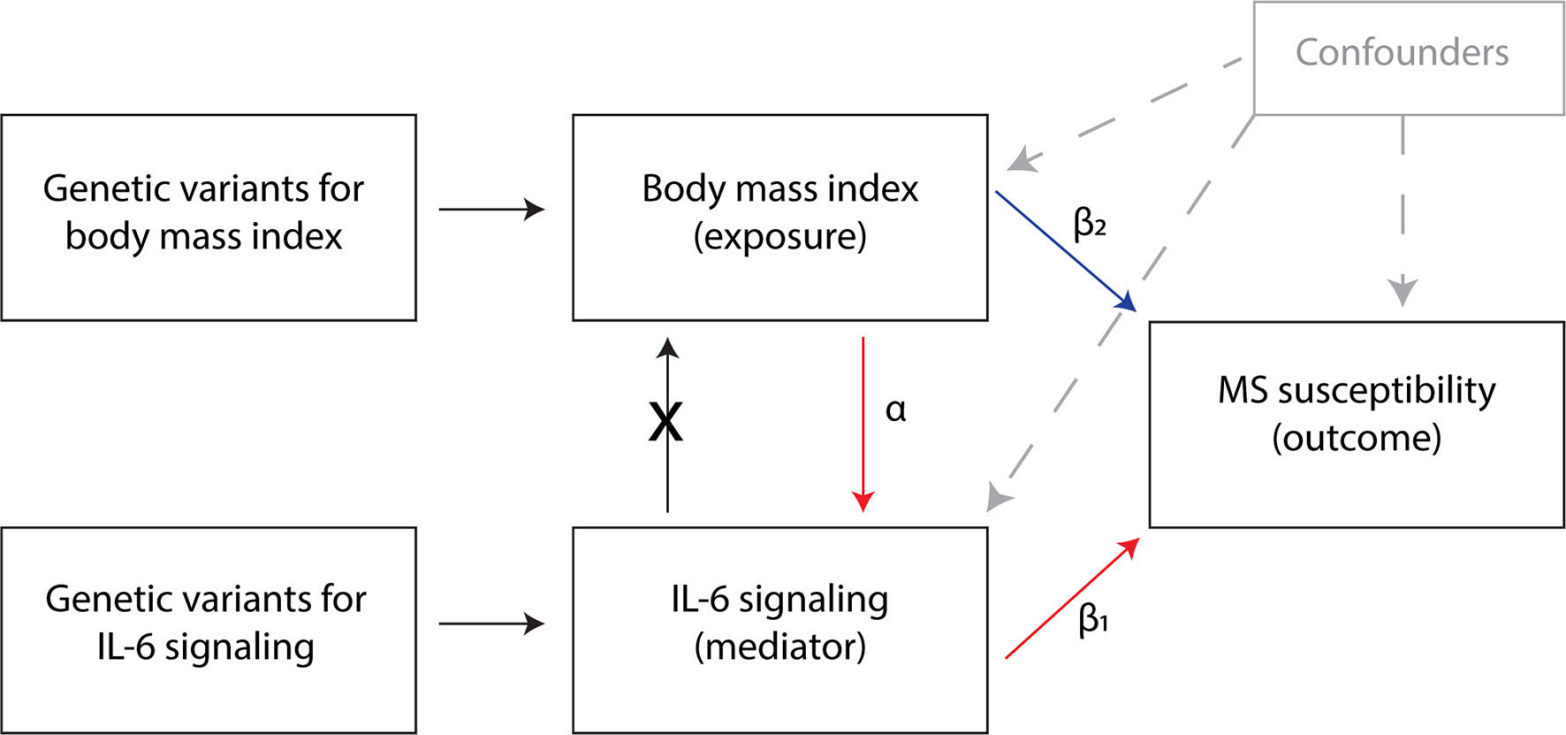
Directed acyclic graph of the Mendelian randomization mediation analysis. α = the effect of BMI on IL-6 signaling levels; β1 = the effect of IL-6 signaling on MS, adjusted for BMI using multivariable MR; β2 = the effect of BMI on MS, independently from IL-6 signaling; the indirect effect is estimated by multiplying α with β1. The proportion mediated is estimated by dividing the indirect effect (α * β_1_) by the total effect of BMI on MS.

Multivariable and mediation analyses were performed with BMI data from Yengo et al. (22) and CRP (proxy for IL-6 signaling) data from the CHARGE Inflammation Working Group (25) or from UK Biobank (24) as exposures, and MS risk as outcome (29). The directed acyclic graph of the MR mediation analysis is depicted in **Figure 1**. The indirect effect estimate was obtained using the product of coefficients method, i.e. by the multiplication of the β coefficients from the univariable MR analysis with BMI as exposure and IL-6 signaling as outcome (α) and from the multivariable MR analysis with IL-6 signaling as exposure and MS as outcome, conditioned on BMI (β_1_). The total effect is obtained from the univariable analysis with BMI as exposure and MS risk as outcome, without adjustment for IL-6 signaling (42). The proportion mediated was estimated by dividing the indirect effect (α * β_1_) by the total effect of BMI on MS (11, 43).

The odds ratios (ORs) and *p* values of the MS risk SNPs were transformed into β coefficients and standard errors for subsequent analyses. As the BMI phenotype in the GIANT meta-analysis was normalized, β coefficients correspond to SD of BMI, with 1 SD equaling a mean of 4.70 BMI units (kg/m^2^) among cohorts in the GIANT consortium (22). For IL-6 signaling SNPs (proxied by CRP) derived from the UK Biobank, β coefficients correspond to a 1-unit change in CRP levels. For IL-6 signaling, CRP and IL-6 SNPs derived from the CHARGE Inflammation Working Group, β coefficients correspond to 1-unit change in natural-log transformed CRP and IL-6.

## Results

### Genetically Predicted BMI and Multiple Sclerosis

**Figure 2:**
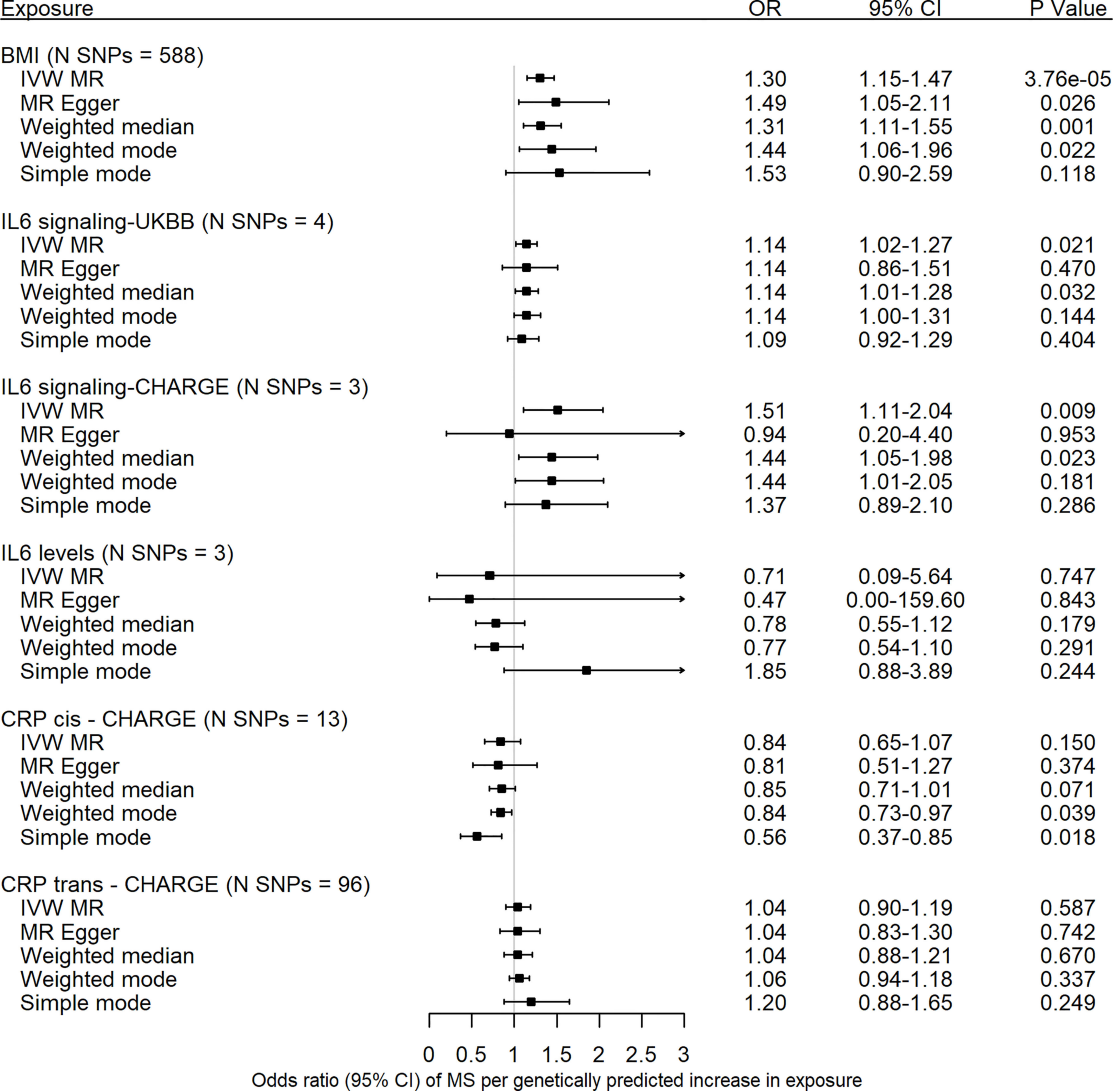
Forest plot of univariable Mendelian randomization estimates of body mass index, interleukin-6 signaling, interleukin-6 levels and c-reactive protein with risk of multiple sclerosis. Data are displayed as odds ratio (OR) and 95% confidence interval (CI) per SD increase in genetically predicted BMI levels, per unit increase in c-reactive protein (CRP) levels for interleukin-6 (IL-6) signaling UKBB, per unit increase in natural-log transformed CRP levels for IL-6 signaling CHARGE and CRP CHARGE, and per unit increase in natural-log transformed IL-6 levels for IL-6 levels CHARGE. UKBB, UK Biobank; CHARGE, Cohorts for Heart and Aging Research in Genomic Epidemiology; CRP, c-reactive protein; MR, Mendelian randomization; N SNPs, number of variants in the analysis; SNP, single nucleotide polymorphisms; IVW, inverse-variance weighted method.

Genetic predisposition to an increased BMI, with BMI SNPs from the latest meta-analysis by Yengo et al. (22), was associated with MS susceptibility (IVW: odds ratio (OR) = 1.30, 95% confidence interval [CI] = 1.15-1.47, *p* = 3.76 × 10^-5^) (**Figure 2**). There was no evidence for directional pleiotropy from the MR Egger regression intercept nor from the funnel plot (**Supplementary Figure 1**), though the Cochran Q test and *I^2^* statistic did show heterogeneity among individual SNP effect estimates (**Supplementary Table 30**). Excluding the MHC SNP rs498240 led to very similar findings (**Supplementary Table 30**). The MR-PRESSO global test *p* was < 0.0001, with eleven SNPs identified as outliers (rs1048932, rs1106908, rs2010281, rs273504, rs3803286, rs3810291, rs3814883, rs419261, rs498240, rs7535528, rs7941030). MR-PRESSO outlier-corrected estimates were consistent with the main IVW analysis (**Supplementary Table 30**). The effect of BMI on MS risk was replicated using a smaller set of 69 variants from an earlier BMI GWAS (23), excluding the UK Biobank cohort (**Supplementary Figure 2** and **Supplementary Table 30**).

### Genetically Predicted IL-6 Signaling, IL-6 Levels, CRP and Multiple Sclerosis

Significant effects were observed for increased IL-6 signaling on risk of MS (IL-6 signaling UK Biobank: OR = 1.14, 95% CI = 1.02-1.27, *p* = 0.02; IL-6 signaling CHARGE: OR = 1.51, 95% CI = 1.11-2.04, *p* = 0.01) (**Figure 2**, **Supplementary Table 31** and **Supplementary Figure 3**). There was no evidence for pleiotropy nor heterogeneity (**Supplementary Table 31**). In contrast to IL-6 signaling, we found little evidence for an effect of serum IL-6 levels on risk of MS (OR = 0.71, 95% CI = 0.09-5.64, *p* = 0.75) (**Figure 2**, **Supplementary Table 31** and **Supplementary Figure 4**). In addition, there was considerable heterogeneity among the individual SNP effect estimates (**Supplementary Table 31**). There was no evidence for pleiotropy from the MR Egger intercept (**Supplementary Table 31**). After exclusion of the MHC SNP rs660895, the IVW estimate was similar as the original analysis, though with a more precise 95% confidence interval (**Supplementary Table 31**).

To disentangle the effect of upregulated IL-6 signaling from the effect of CRP, we next performed MR analyses to explore associations between SNPs associated with CRP and MS risk. Both cis- and trans MR analyses showed little evidence for an association between genetically determined CRP levels and risk of MS (**Figure 2**, **Supplementary Table 32** and **Supplementary Figure 5**).

In bi-directional MR analyses, genetically predicted MS risk as exposure was not associated with BMI (**Supplementary Table 33**), neither with IL-6 signaling or serum IL-6 levels (**Supplementary Table 34** and **Supplementary Figure 6** and **Supplementary Figure 7**).

### Genetically Predicted BMI and IL-6 Signaling and IL-6 Serum Levels

Significant effects were observed for genetically predicted BMI (22) on IL-6 signaling and serum IL-6 levels (IL-6 signaling UK Biobank: β = 0.99, 95% CI = 0.88,1.09, *p* = 1.56 × 10^-74^; IL-6 signaling CHARGE: β = 0.37, 95% CI = 0.32,0.41, *p* = 1.58 × 10^-65^, IL-6 levels CHARGE: β = 0.15, 95% CI = 0.12,0.18, *p* = 4.81 × 10^-25^) (**Supplementary Table 35** and **Supplementary Figure 8**). There was no evidence for pleiotropy from the MR Egger intercept test, except for IL-6 signaling data from the CHARGE Inflammation Working Group as outcome (**Supplementary Table 35**). Heterogeneity amongst individual SNP effect estimates was present in all three analyses (**Supplementary Table 35**). Exclusion of the MHC SNP rs498240 led to similar effect estimates (**Supplementary Table 35**). Likewise, MR-PRESSO outlier corrected estimates were in line with those obtained in the main IVW analyses (**Supplementary Table 35**).

Potentially, there is sample overlap between the latest BMI dataset (22) and the IL-6 signaling dataset from the UK Biobank. Therefore, secondary analyses were performed with the BMI dataset from Locke et al. (23), not including UK Biobank data. Effect estimates were in the same direction and order of magnitude as our primary analysis with the latest BMI dataset (22) (**Supplementary Table 36**).

In contrast, genetically predicted increased IL-6 signaling did not influence BMI (IL-6 signaling CHARGE: β = -0.004, 95% CI = -0.057,0.049, *p* = 0.87) (**Supplementary Table 37**).

### Mediation of the Effect of Genetically Predicted BMI on MS by IL-6 Signaling

**Figure 3:**
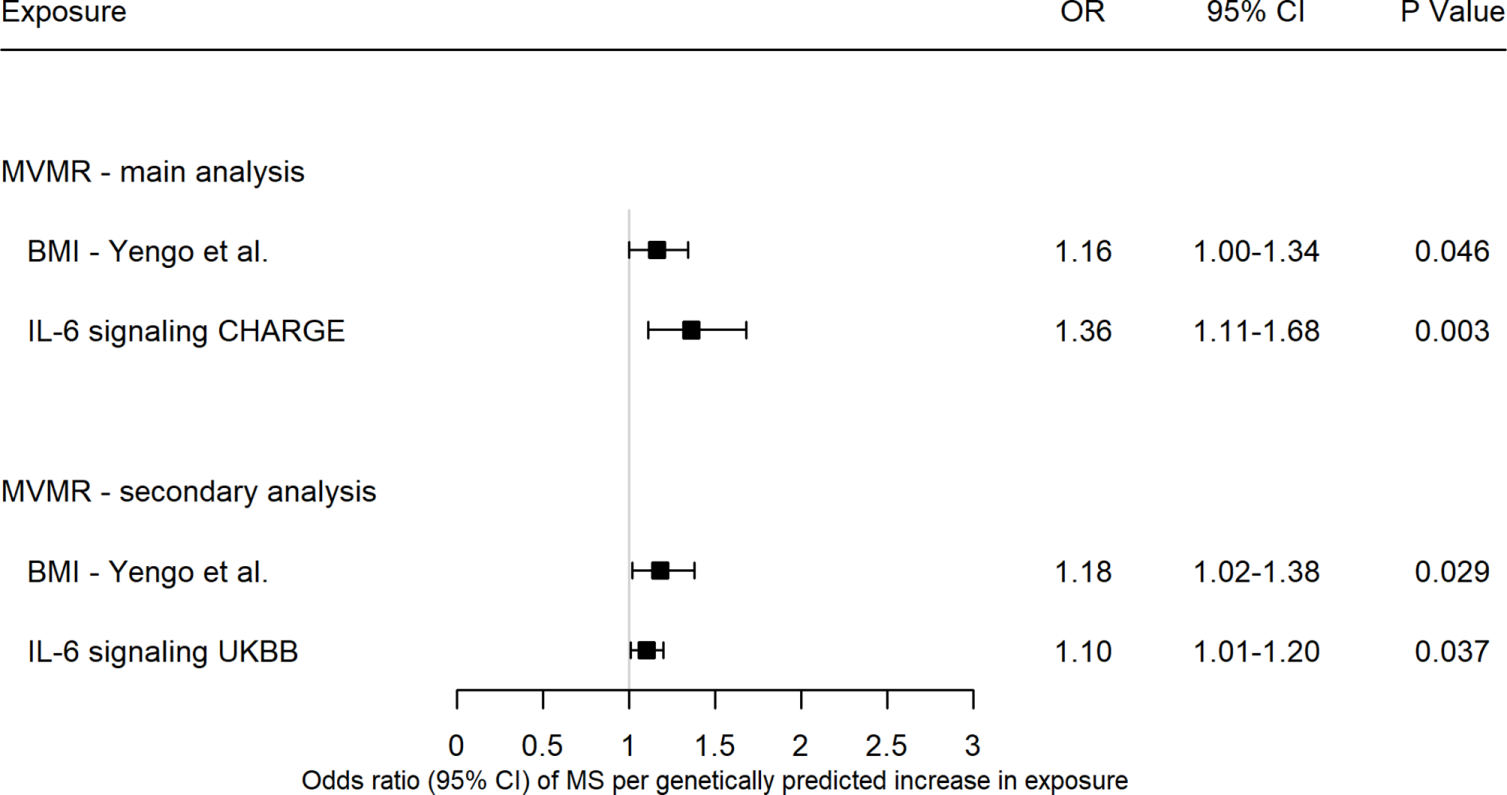
Forest plot of multivariable Mendelian randomization estimates from the inverse-variance weighted method of body mass index and interleukin-6 signaling with risk of multiple sclerosis. Data are displayed as odds ratio (OR) and 95% confidence interval (CI) per SD increase in genetically predicted body mass index (BMI) levels, per unit increase in c-reactive protein (CRP) levels for interleukin-6 (IL-6) signaling UKBB, per unit increase in natural-log transformed CRP levels for IL-6 signaling CHARGE. BMI, body mass index; UKBB, UK Biobank; CHARGE, Cohorts for Heart and Aging Research in Genomic Epidemiology; MVMR, multivariable Mendelian randomization.

In multivariable MR analyses with IL-6 signaling data from the CHARGE consortium, the effect of IL-6 signaling on MS remained after adjusting for BMI (OR = 1.36, 95% CI = 1.11-1.68, *p* = 0.003), with smaller effect estimate and confidence intervals after adjustment (**Figure 3**). Likewise, the effect of higher BMI on MS after adjustment for IL-6 signaling remained, though this direct effect is considerably smaller in effect size and only borderline significant compared to the total effect obtained in the univariable analyses without adjustment for IL-6 signaling (MVMR: OR = 1.16, 95% CI =1.00-1.34, *p* = 0.046) (**Figure 3**). Results from methods robust to pleiotropy (MVMR-Egger, MVMR-Median, MVMR-Robust and MVMR-Lasso) and weak instruments (MVMR-Q(het)) for multivariable Mendelian randomization (40, 41) are depicted in **Supplementary Figure 9**. These methods gave results which were broadly consistent with the MVMR-IVW results. The MVMR-Egger, MVMR-median and MVMR-Q(het) methods suggested a null causal effect of BMI on risk of MS, however their point estimates are still in the same direction and all other methods were in line with MVMR-IVW. The proportion of the effect of BMI on MS, mediated by IL-6 signaling was estimated by dividing the indirect effect by the total effect of BMI on MS and corresponded to 43% (95% CI = 25%-54%). As secondary analysis, using the UK Biobank data for IL-6 signaling, the proportion of the effect of BMI on MS corresponded to 35%, albeit with a larger 95% confidence interval (95% CI = 3%-51%).

## Discussion

In this MR study, we investigated the role of interleukin-6 signaling in mediating the effect of BMI on risk of MS. First, our results show evidence for a causal role of genetically predicted increased BMI and upregulated IL-6 signaling in the development of MS. Second, genetically predicted increased BMI is associated with upregulated IL-6 signaling. In contrast, genetically predicted increase in IL-6 signaling did not influence BMI. Finally, considering both BMI and interleukin-6 signaling in a Mendelian randomization mediation analysis, approximately 40% of the effects of body mass index on the development of MS are mediated through interleukin-6 signaling.

Our findings are consistent with observational studies and studies applying a polygenic risk score approach and further support an effect of BMI on risk of MS (4–6, 8–12) and of BMI on IL-6 levels (19, 44–47). For the first time, we leveraged large-scale genetic associations within the two-sample MR paradigm to investigate the causal role of BMI on IL-6 signaling and serum levels. Two prior MR studies have investigated the relation between IL-6 and risk of MS. Zhang et al. demonstrated a causal role between increased soluble IL-6R levels and decreased risk of MS (48). These results are in line with our findings as increased soluble IL-6R levels reflect reduced IL-6 signaling *via* the classical pathway. In contrast, Lu et al. found little evidence for a role of serum IL-6 levels in the risk of MS (49), again in line with our results. A potential explanation for the discrepancy between IL-6 signaling and IL-6 levels may be weak instrument bias, as the instruments selected for serum IL-6 levels explain solely ~1% of phenotypic variance (28) while the instruments selected for IL-6 signaling explain ~5% of phenotypic variation (26).

We applied a cis-MR approach by selecting genetic proxies for IL-6 receptor mediated regulation of IL-6 signaling within a region of 300kb of the *IL-6R* gene, that were associated with CRP levels. While CRP is a well-established downstream biomarker for IL-6 signaling, and the variants selected reflect higher levels of CRP and thus increased IL-6 signaling, there is little evidence for a causal effect of total CRP levels on risk of MS, in concordance with previous studies (6). This may reflect that the effects of IL-6 signaling on risk of MS are independent of the effects of CRP. In addition, the IL-6 signaling pathway is complex with three different signaling modes, i.e. classic (*via* the membrane-bound IL-6R), trans (*via* the soluble IL-6R) and cluster (IL-6 bound to IL-6R on the surface of dendritic cells presented to T-cells), release of IL-6 from different sources, and pleiotropic effects of IL-6 on different cell types (50). Subsequently, disentangling which IL-6 signaling modes and cell types are involved needs further clarification which goes beyond the scope of this study. Studies are now emerging that implement the MR design to better understand the pleiotropic nature of IL-6 signaling. Recently, Rahman and colleagues leveraged genetic variants that proxy IL-6 signaling to investigate effects on levels of circulating cytokines, chemokines and growth factors (51). Among considered cytokines, increased IL-6 signaling was associated with reduced levels of interferon-ɣ, interleukin-4, interleukin-10 and interleukin-12 (51). Besides MR studies, *in vitro* studies in T-cells from MS patients showed that blockade of IL-6 signaling by anti-IL-6R monoclonal antibody reduces IL-17 production and elevates IL-10 release by activated CD4+ T cells (52). A deleterious role of IL-6 signaling in MS has been further demonstrated in studies in experimental autoimmune encephalomyelitis (EAE) mice where IL-6R blockade prevents the development of EAE in IL-6 sufficient mice (53) and IL-6 ^-/-^ mice are resistant to EAE (54, 55). Transfer of autoantigen-loaded IL-6 sufficient dendritic cells renders IL-6-deficient mice fully susceptible to EAE, pointing towards dendritic cells as a key early source of IL-6 (56). Altogether, these findings further provide insights into the effects of IL-6 signaling.

Our study has a number of strengths. We minimized pleiotropy by choosing genetic variants in the proximity to the *IL-6R* gene that are associated with CRP, which is a reliable biomarker of IL-6R signaling. Furthermore, the MR-Egger intercept did not suggest biasing pleiotropy, and results were consistent across methods more robust to pleiotropy, such as MR-Egger, weighted median, weighted mode and MR-PRESSO. Finally, we performed sensitivity analyses excluding SNPs within the MHC region to minimize bias related to pleiotropic effects of MHC SNPs. Likewise, we extended our multivariable analyses to methods robust to pleiotropy and weak instruments.

We also acknowledge a number of limitations. First, as individuals of European ancestry only were included in the GWASs from which we selected our instrumental variables, the generalization of our findings to other populations is limited. Second, biological compensation can occur, i.e. genetically proxied IL-6 signaling may be compensated by changes in other pathways. Third, the results from the MR analyses reflect life-long effects of the instrumental variables on IL-6 signaling and may therefore not immediately be extrapolated to estimate the magnitude of effect of clinical interventions. Fourth, the non-collapsibility of OR can cause biased mediation estimates. However, this is lessened by the use of the product of coefficients method (57). Fifth, inherent to our threshold for clumping (*r^2^* of 0.05) genetic instruments might still be correlated. Hence, this may lead to an overestimation of the true causal effects. Finally, although our findings do not provide evidence for pleiotropic bias, we cannot entirely rule out the possibility that genetic proxies for IL-6 signaling affect other pathways unrelated than those of IL-6R signaling.

Previously, it has been shown that lowered vitamin D levels mediate only a small proportion of the effect of obesity on risk of MS (11). Taken together with our current findings, vitamin D and interleukin-6 signaling explain approximately 50% of the association between obesity and MS susceptibility. Despite the identification of IL-6 signaling as major mediator, still half of the association between BMI and MS susceptibility remains unexplained. Further explorations of pathways underlying the association between BMI and MS are required. These include, but are not limited to, the investigation of other inflammatory adipokines and changes in metabolites and composition of gut microbiota. These findings will improve our understanding of MS biology and potentially lead to improved opportunities for targeted prevention strategies.

## Data Availability Statement

The datasets presented in this study can be found in online repositories. The names of the repository/repositories and accession number(s) can be found in the article/**Supplementary Material**.

## Author Contributions

MV, BD, and AG contributed to the conception and design of the study. MV and SB performed the statistical analysis. MV wrote the first draft of the manuscript. All authors contributed to the article and approved the submitted version.

## Funding

MV is a PhD Fellow (11ZZZ21N) and BD a Clinical Investigator of the Research Foundation-Flanders (FWO-Vlaanderen). AG and BD received support from the Research Fund KU Leuven (C24/16/045), the Research Foundation-Flanders (FWO G.07334.15), the Belgian Charcot Foundation and MS Liga Vlaanderen. The computational resources and services used in this work were provided by the VSC (Flemish Supercomputer Center), funded by the Research Foundation-Flanders (FWO-Vlaanderen) and the Flemish Government (department EWI). AG and BD received support from the Horizon2020 “MultipleMS” consortium (grant EU RIA 733161).

## Conflict of Interest

BD has received consulting fees and/or funding from Biogen Idec, BMS, Sanofi-Aventis and Teva. AG and BD have received consulting fees, travel funding and/or research funding from Roche, Novartis and Merck.

The remaining authors declare that the research was conducted in the absence of any commercial or financial relationships that could be construed as a potential conflict of interest.

## Publisher’s Note

All claims expressed in this article are solely those of the authors and do not necessarily represent those of their affiliated organizations, or those of the publisher, the editors and the reviewers. Any product that may be evaluated in this article, or claim that may be made by its manufacturer, is not guaranteed or endorsed by the publisher.
