# Supplementary material for: Body Mass Index, Interleukin-6 Signaling and Multiple Sclerosis: A Mendelian Randomization Study
Source: Front Immunol. 2022 Mar 21;13:834644. doi: 10.3389/fimmu.2022.834644 (PMC8978959; doi:10.3389/fimmu.2022.834644)
Supplement: Supplementary file 1 [file DataSheet_1.pdf]

Supplementary Material

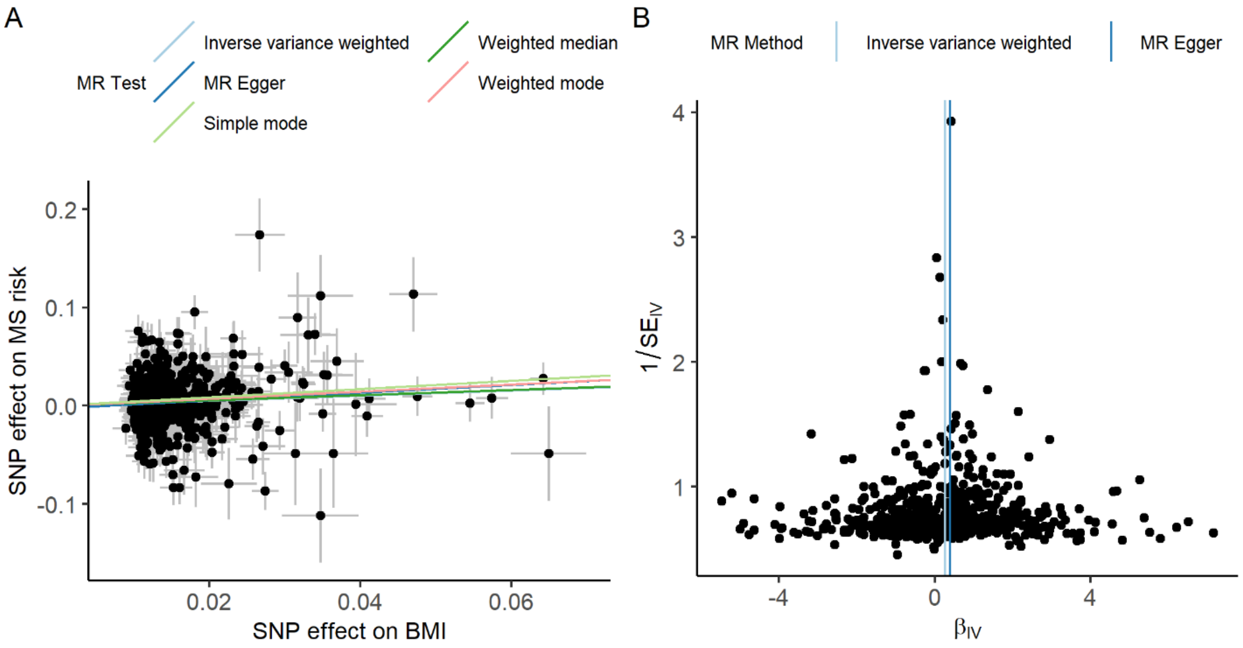

Supplementary Figure 1 Scatter plot and funnel plot of the relation between body mass index and risk of multiple sclerosis, with genetic variants for body mass index derived from Yengo et al study (PMID 30124842). **A.** For each single-nucleotide polymorphism (SNP), the effect estimates on exposure, genetically predicted body mass index (BMI) levels, and on outcome, risk of multiple sclerosis (MS), are plotted on log-odds ratio scale. The slope of the lines corresponds to the estimated causal effect of genetically predicted BMI on risk of MS. **B.** For each SNP, the resulting Mendelian randomization (MR) estimate is plotted against the inverse of the standard error of the MR estimate. Symmetry noted in this plot provides evidence against the presence of horizontal pleiotropy. The vertical lines represent the summary measure of the effect of BMI on risk of MS on the log-odds ratio scale from inverse-variance weighted (IVW) and MR Egger tests

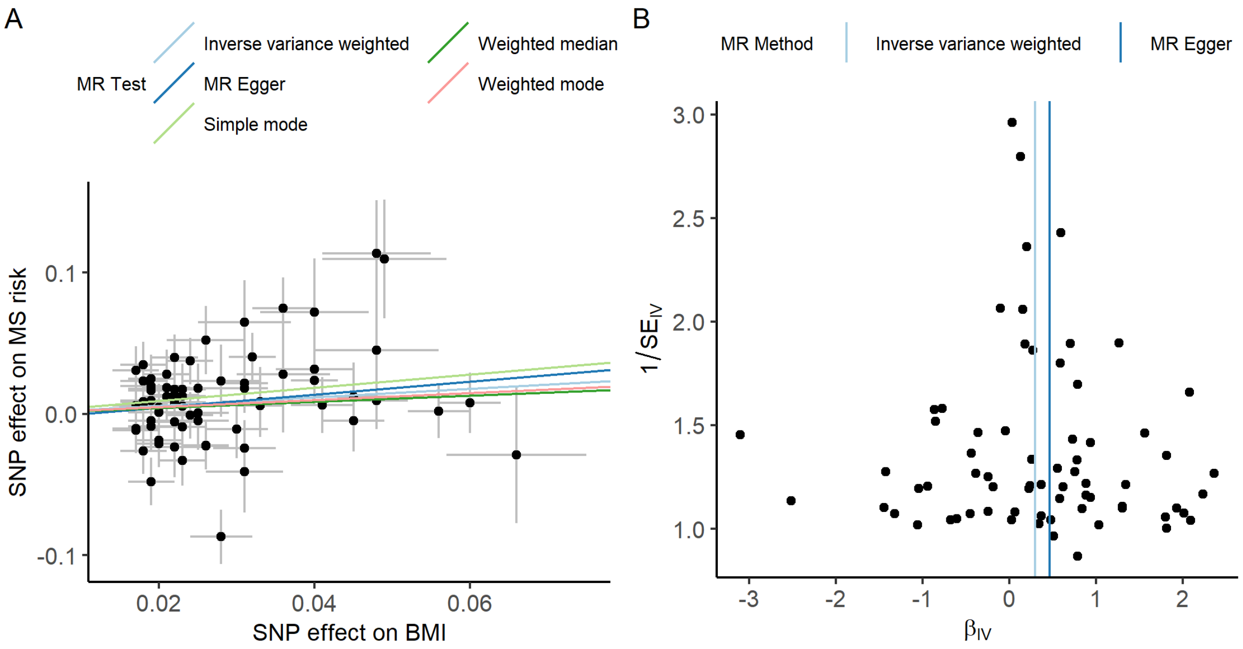

Supplementary Figure 2 Scatter plot and funnel plot of the relation between body mass index and risk of multiple sclerosis, with genetic variants for body mass index derived from Locke et al study (PMID 25673413). **A.** For each single-nucleotide polymorphism (SNP), the effect estimates on exposure, genetically predicted BMI levels, and on outcome, risk of MS, are plotted on log-odds ratio scale. The slope of the lines corresponds to the estimated causal effect of genetically predicted BMI on risk of MS. **B.** For each SNP, the resulting Mendelian randomization (MR) estimate is plotted against the inverse of the standard error of the MR estimate. Symmetry noted in this plot provides evidence against the presence of horizontal pleiotropy. The vertical lines represent the summary measure of the effect of BMI on risk of MS on the log-odds ratio scale from inverse-variance weighted (IVW) and MR Egger tests

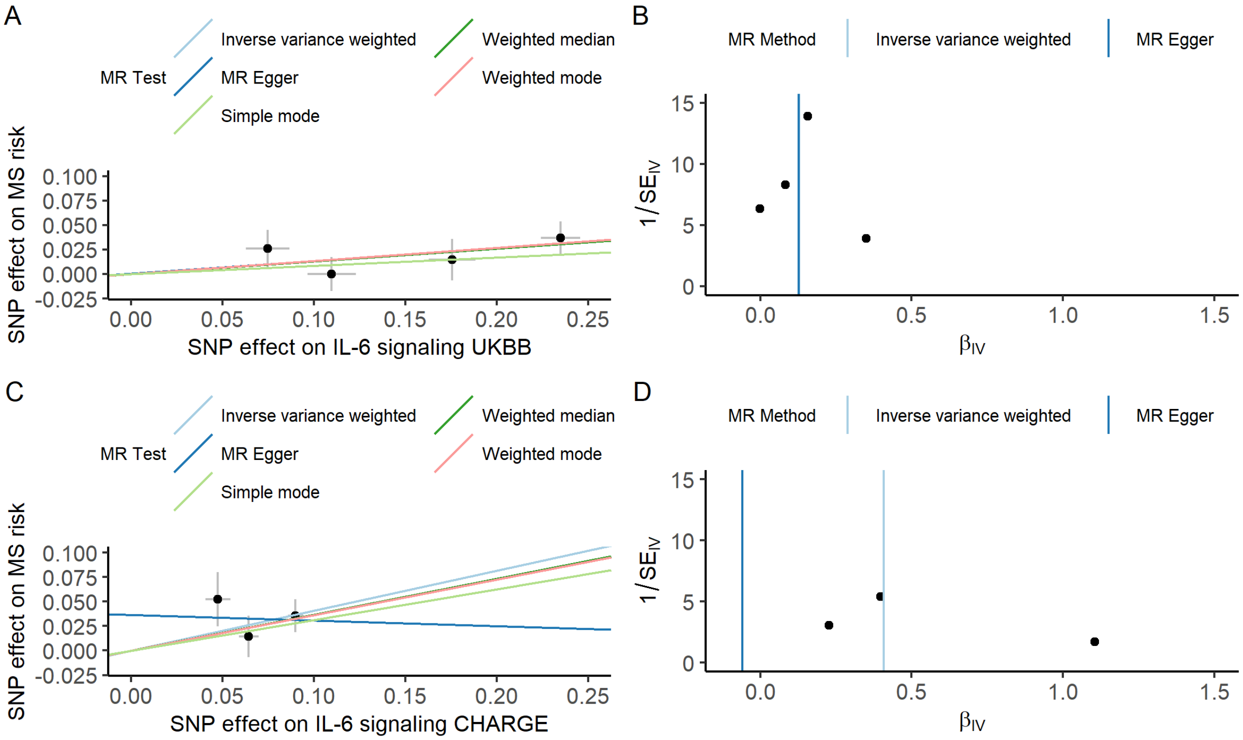

Supplementary Figure 3 Scatter plots and funnels plot of the relation between interleukin-6 signaling and risk of multiple sclerosis. **A.** Scatter plot with genetically predicted IL-6 signaling from the UK Biobank GWAS for CRP as exposure and risk of MS as outcome **B.** Funnel plot of the Mendelian randomization (MR) estimate plotted against the inverse of the standard error of the MR estimate from the univariable analysis with genetically predicted IL-6 signaling from the UK Biobank GWAS for CRP as exposure and risk of MS as outcome **C.** Scatter plot with genetically predicted IL-6 signaling from the CHARGE GWAS for CRP as exposure and risk of MS as outcome **D.** Funnel plot of the MR estimate plotted against the inverse of the standard error of the MR estimate from the univariable analysis with genetically predicted IL-6 signaling from the CHARGE GWAS for CRP as exposure and risk of MS as outcome

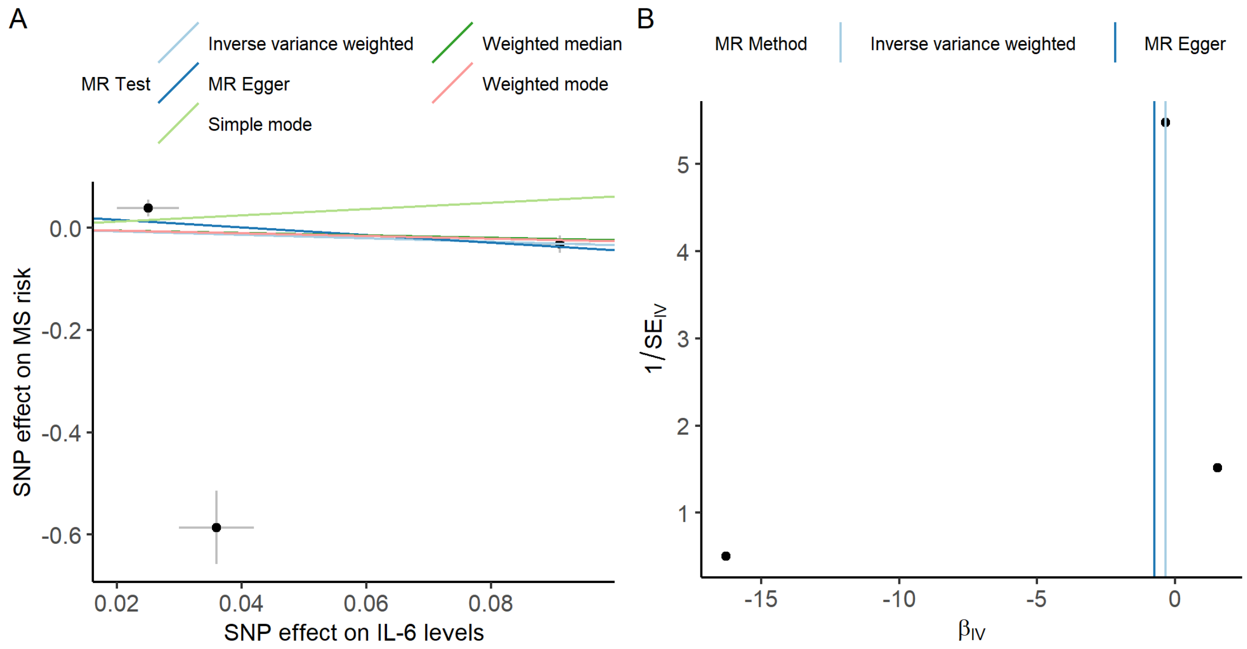

Supplementary Figure 4 Scatter plot and funnel plot of the relation between interleukin-6 levels and risk of multiple sclerosis **A.** For each single-nucleotide polymorphism (SNP), the effect estimates on exposure, genetically predicted IL-6 levels, and on outcome, risk of MS, are plotted on log-odds ratio scale. The slope of the lines corresponds to the estimated causal effect of genetically predicted IL-6 levels on risk of MS. **B.** For each SNP, the resulting Mendelian randomization (MR) estimate is plotted against the inverse of the standard error of the MR estimate. Symmetry noted in this plot provides evidence against the presence of horizontal pleiotropy. The vertical lines represent the summary measure of the effect of IL-6 levels on risk of MS on the log-odds ratio scale from inverse-variance weighted (IVW) and MR Egger tests

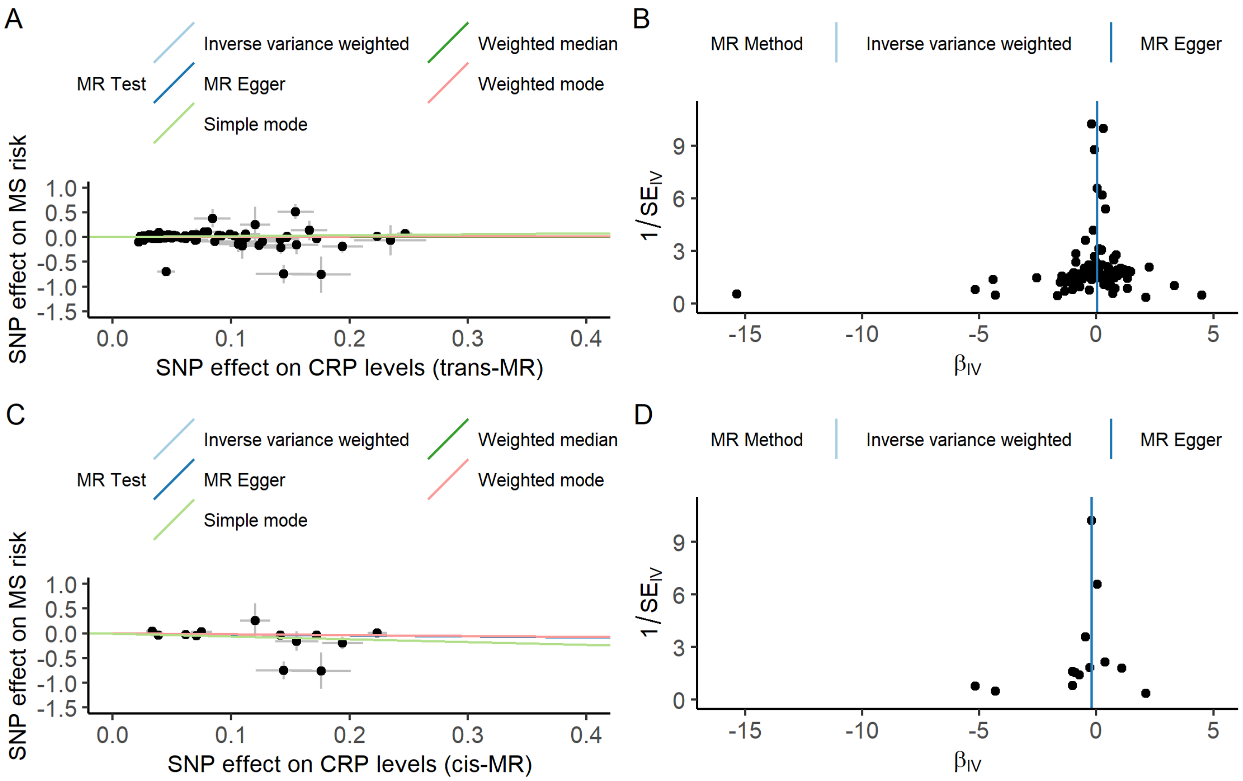

Supplementary Figure 5 Scatter plots and funnels plot of the relation between C-reactive protein levels and risk of multiple sclerosis. **A.** Scatter plot with genetically predicted CRP levels from CHARGE as exposure and risk of MS as outcome in trans-MR analysis **B.** Funnel plot of the trans Mendelian randomization (MR) estimate plotted against the inverse of the standard error of the trans-MR estimate from the univariable analysis with genetically predicted CRP levels as exposure and risk of MS as outcome **C.** Scatter plot with genetically predicted CRP levels from CHARGE as exposure and risk of MS as outcome in cis-MR analysis **D.** Funnel plot of the cis Mendelian randomization (MR) estimate plotted against the inverse of the standard error of the cis-MR estimate from the univariable analysis with genetically predicted CRP levels as exposure and risk of MS as outcome

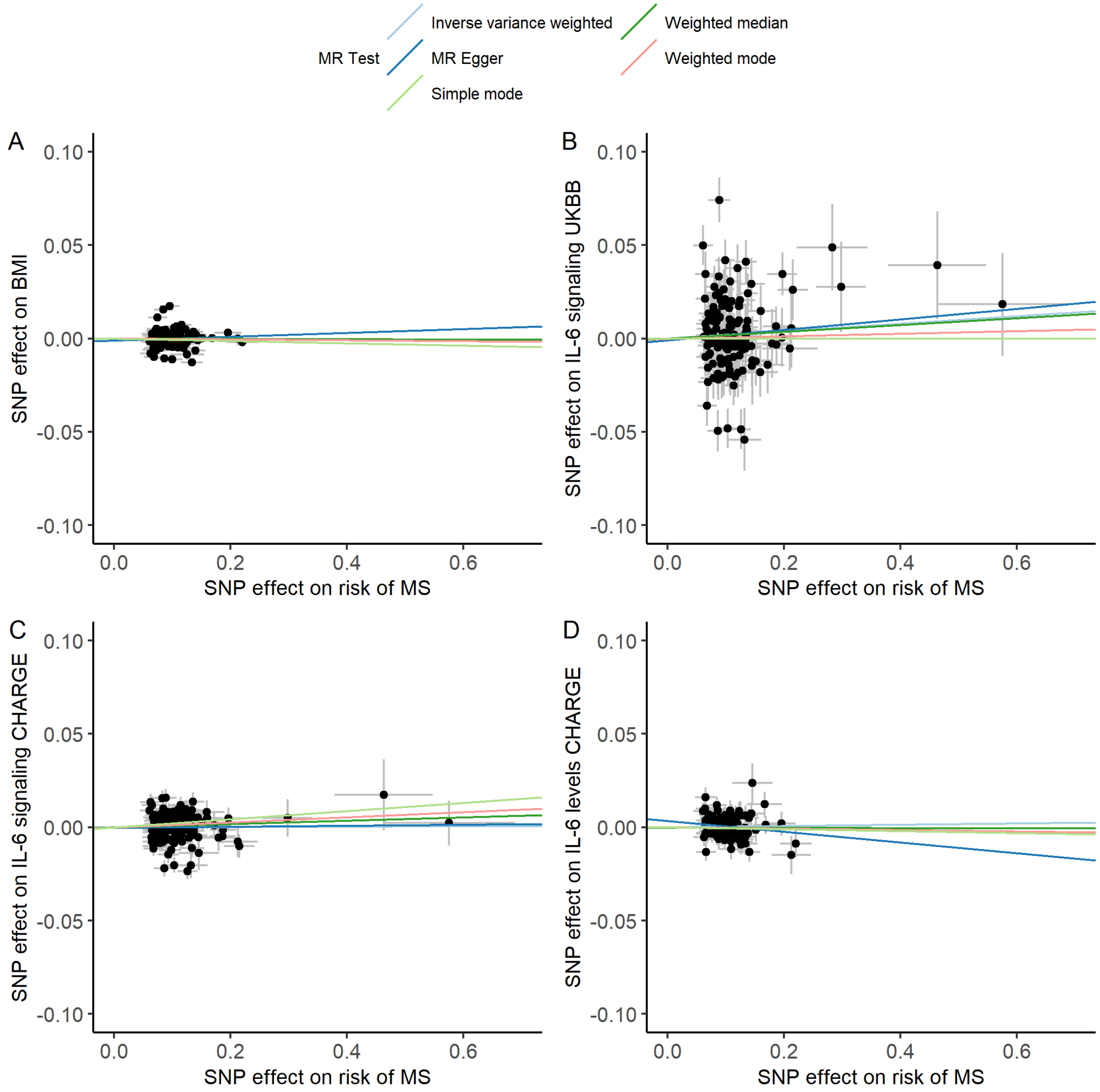

Supplementary Figure 6. Scatter plots of the relation between multiple sclerosis risk as exposure and body mass index, interleukin-6 signaling and interleukin-6 levels as outcome **A**. Genetically predicted BMI with estimates derived from Yengo et al GWAS (PMID 30124842) as outcome **B**. Genetically predicted IL-6 signaling as outcome with estimates from the UK Biobank GWAS for CRP **C.** Genetically predicted IL-6 signaling as outcome with estimates from the CHARGE GWAS for CRP **D.** Genetically predicted IL-6 levels as outcome with estimates from the CHARGE GWAS for IL-6 levels.

frontiers

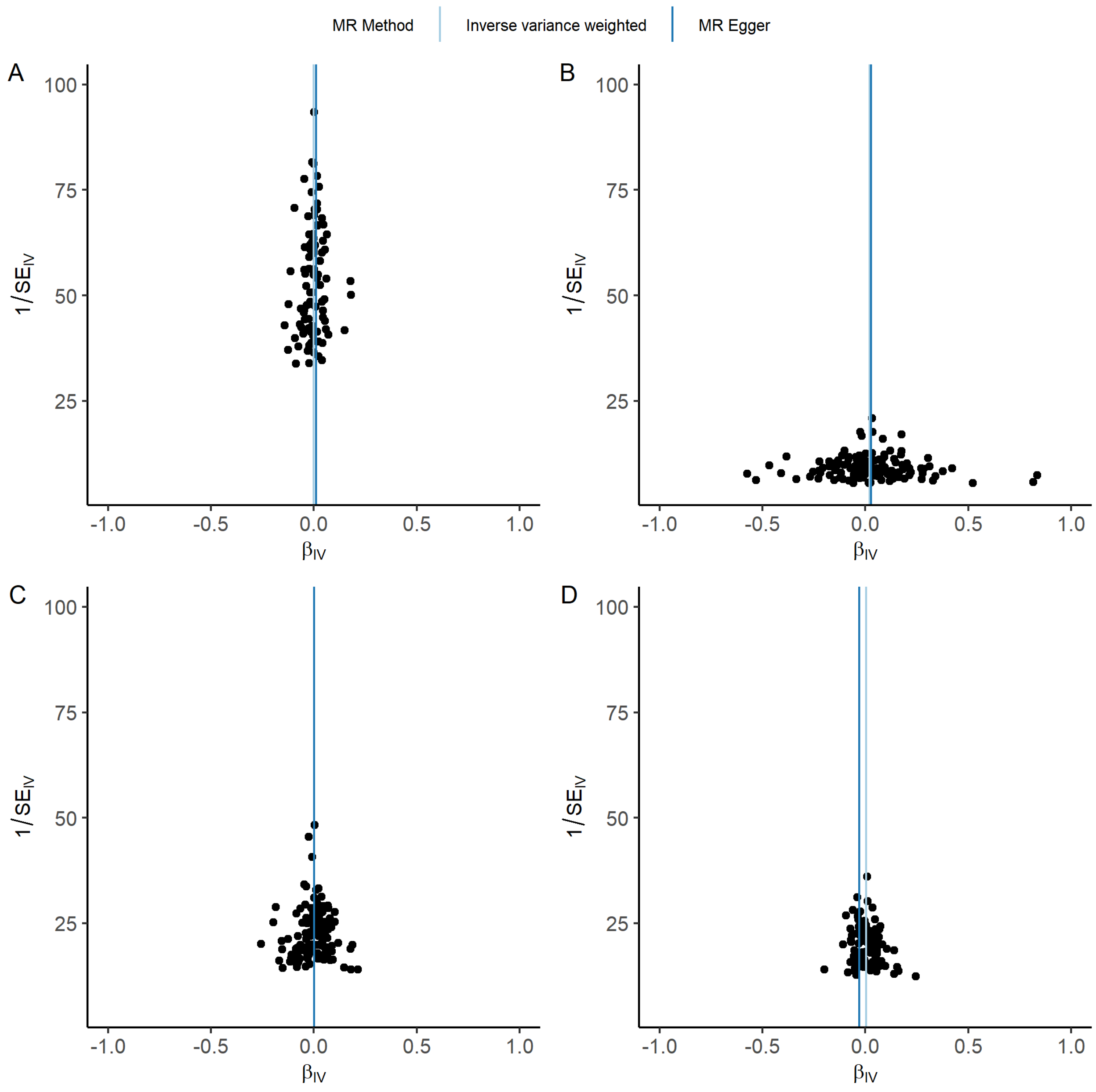

Supplementary Figure 7. Funnel plots of the Mendelian randomization analyses with multiple sclerosis risk as exposure and body mass index, C-reactive protein and interleukin-6 levels as outcome **A**. Genetically predicted BMI with estimates derived from Yengo et al GWAS (PMID 30124842) as outcome **B**. Genetically predicted IL-6 signaling as outcome with estimates from the UK Biobank GWAS for CRP **C.** Genetically predicted IL-6 signaling as outcome with estimates from the CHARGE GWAS for CRP **D.** Genetically predicted IL-6 levels as outcome with estimates from the CHARGE GWAS for IL-6 levels.

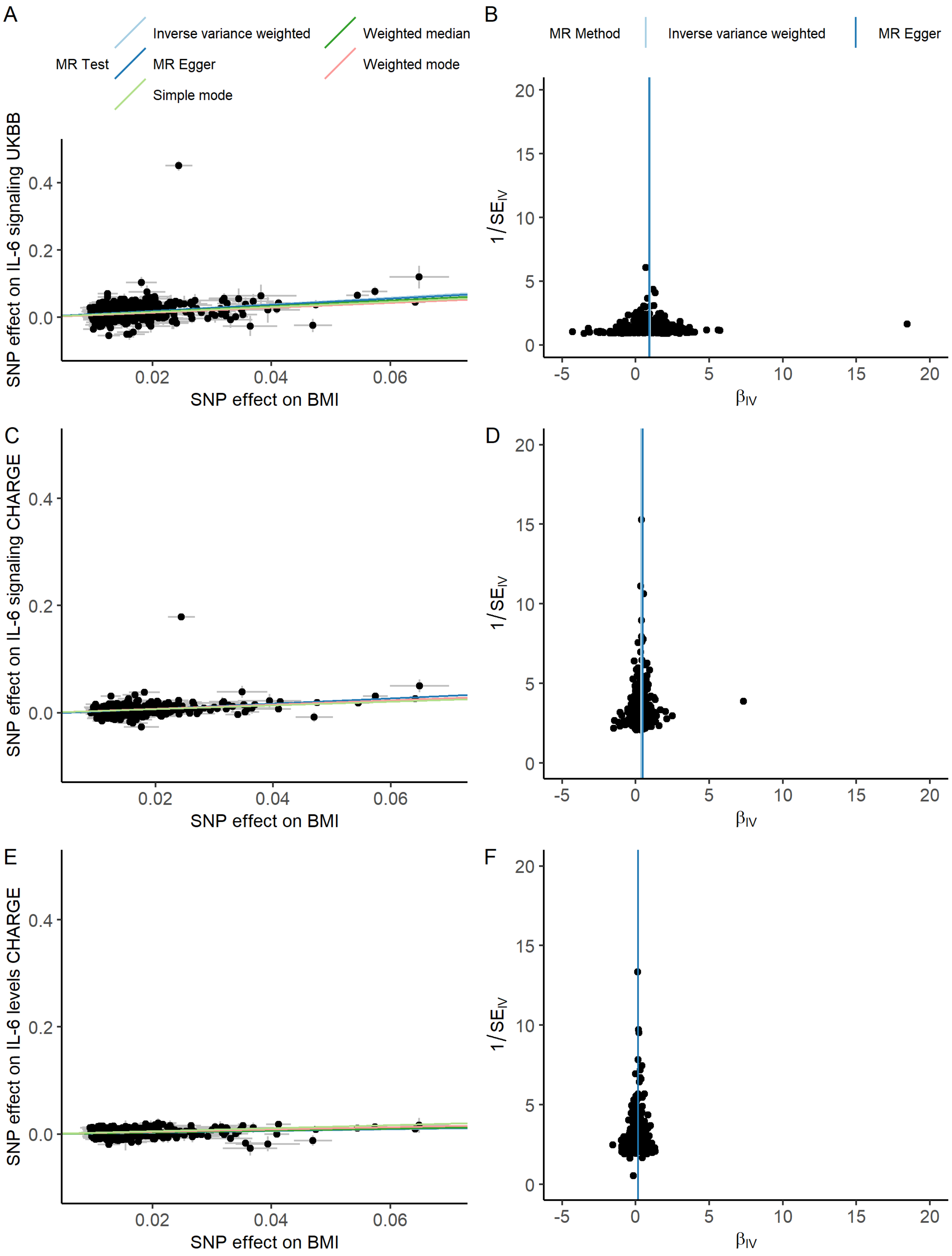

A
MR Test
Inverse variance weighted
MR Egger
Simple mode
Weighted median
Weighted mode
SNP effect on IL-6 signaling UKBB
SNP effect on BMI
B
MR Method
Inverse variance weighted
MR Egger
1/SE_IV
β_IV
C
SNP effect on IL-6 signaling CHARGE
SNP effect on BMI
D
1/SE_IV
β_IV
E
SNP effect on IL-6 levels CHARGE
SNP effect on BMI
F
1/SE_IV
β_IV

Supplementary Figure 8 Scatter plots and funnels plot of the Mendelian randomization analyses with body mass index as exposure and interleukin-6 signaling or interleukin-6 -6 levels as outcome. **A.** Scatter plot with genetically predicted BMI with estimates derived from Yengo et al GWAS (PMID 30124842) as exposure and genetically predicted IL-6 signaling from the UK Biobank GWAS for CRP as outcome **B.** Funnel plot of the Mendelian randomization (MR) estimate plotted against the inverse of the standard error of the MR estimate from the univariable analysis with genetically predicted BMI levels as exposure and IL-6 signaling from the UK Biobank GWAS for CRP as outcome **C.** Scatter plot with genetically predicted BMI with estimates derived from Yengo et al GWAS (PMID 30124842) as exposure and genetically predicted IL-6 signaling from the CHARGE GWAS for CRP as outcome **D.** Funnel plot of the Mendelian randomization (MR) estimate plotted against the inverse of the standard error of the MR estimate from the univariable analysis with genetically predicted BMI levels as exposure and IL-6 signaling from the CHARGE GWAS for CRP as outcome **E.** Scatter plot with genetically predicted BMI as exposure and genetically predicted IL-6 levels from the CHARGE GWAS **F.** Funnel plot of the Mendelian randomization (MR) estimate plotted against the inverse of the standard error of the MR estimate from the univariable analysis with genetically predicted BMI levels as exposure and IL-6 levels from the CHARGE GWAS outcome

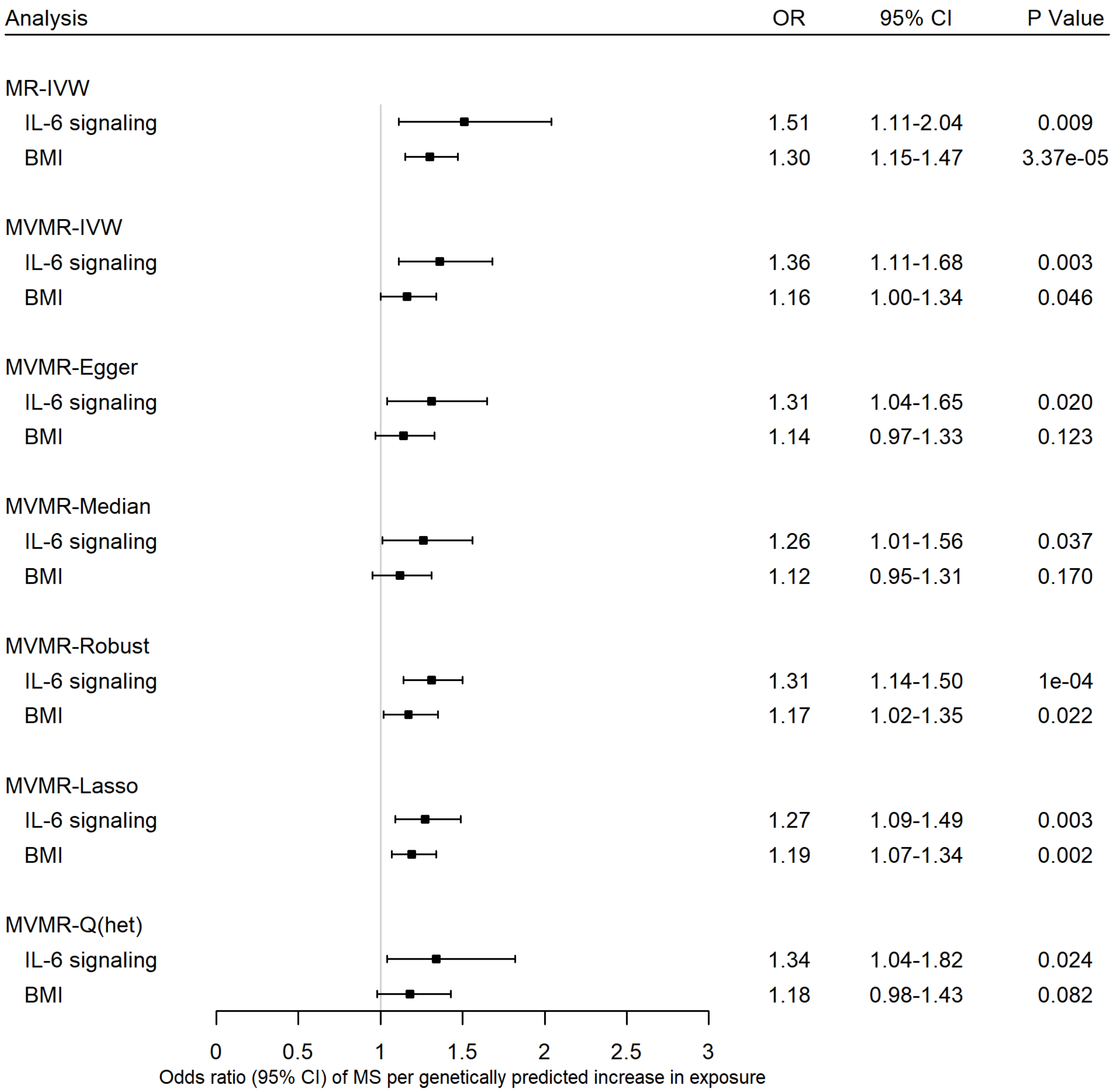

Supplementary Figure 9. Forest plot of univariable and multivariable MR estimates of BMI and IL-6 signaling as exposures and risk of MS as outcome. Data are displayed as odds ratio (OR) and 95% confidence interval (CI) per SD increase in genetically predicted BMI levels and per unit increase in natural-log transformed CRP levels for IL-6 signaling MR = Mendelian randomization; MVMR = multivariable Mendelian randomization; IVW = inverse-variance weighted method
